# Tocilizumab therapy in systemic juvenile idiopathic arthritis – lessons of real clinical practice

**DOI:** 10.1186/1546-0096-12-S1-P67

**Published:** 2014-09-17

**Authors:** Maria I Kaleda, Irina Nikishina, Svetlana Rodionovskaya, Anna Shapovalenko

**Affiliations:** 1 Pediatrics, "Nasonova Research Institute of Rheumatology” , Moscow, Russian Federation

## Introduction

Clinical trials of tocilizumab(TCZ) have verified its efficacy and safety in systemic juvenile idiopathic arthritis (sJIA), but there are many outstanding issues, relevant to the real clinical practice.

## Objectives

to investigate TCZ in pts with sJIA depending on the age of disease onset,duration of disease, the number of active joints, the number of systemic features and previous biologics(B).

## Methods

In prospective study were included 45 pts(19b/26g) with sJIA refractory to conventional treatment, who was treated by TCZ from 9 months to 54 months. At baseline mean age of 6.25(2.0-17.8)yrs; mean disease duration of 4.5(0.3-15.9)yrs. TCZ used as the 1^st^ B in 28 pts, 2^nd^ – in 12, 3^rd^ – in 5. 37.8% pts previously received B:TNF-ingibitors-17, abatacept–2, rituximab–3. 41 pts(91.1%) had arthritis at the baseline. Systemic features were observed in 40(88.9%)pts. Mean number of systemic manifestations(NSM) was 2.82 (1.5;6). 2 pts had MAS before of TCZ initiation. Retrospectively all pts were separated into the groups depending on the age of manifestation of disease (before 3 yrs/older 3 yrs -22/23), duration of disease (less than 3 yrs/more – 20/25), the number of active joints (less than 10/more–22/23), the number of active systemic features (NSM 0 – 13 pts, 1/2 - 10, 3/4 - 12, 5/6 – 10), and previous B (B-naïve/previous B-28/17). Efficacy of TCZ therapy was evaluated in accordance to ACRpedi criteria in 1, 3, 6, 9, 12 and every 6 months of treatment later.

## Results

36 pts continue the treatment, mean duration 27.4 months(9;54). In 9 pts TCZ was cancelled due to serious adverse effects(5), another reasons(4). All pts achieved more than 30-50% improvement by ACRpedi criteria. We found no significant differences in efficacy parameters at the response to therapy depending on the investigated factors. However, pts who had early manifestation of disease, long disease duration, large NSM achieved good response on arthicular status more slowly. Also we observed some increasing of disease activity in all groups between 30-36 months of therapy. Steady improvement allowed to decrease prednisolone (PR) dose in all pts, to cancel PR in 19.4% pts, to cancel NSAIDs in 14% pts. 9 pts achieved inactive disease status. Adverse events were observed independently of investigated factors and included postinfusion reactions (vomiting-1, headache-2, sore throat-4, chestpain-4, blood pressure increasing-1, eczema-2), infections (upper respiratory infections-16, bronchitis-1, pneumonia-2, gastroenteritis-1, ears’ infections-3, periodontitis-2), temporary laboratory abnormalities (neutropenia-21, thrombocytopenia-8, IgG decreasing -10, hyperbilirubinemia-3, elevated transaminases-7). We have observed some SAE: infusion reaction (5); severe infections – varicella (2), atypical pneumonia (1), tuberculosis(1). MAS observed in 4 pts (1-after respiratory infection, 1-after elective surgery, 1-after increase interval between infusion more than 4 weeks, 1-unknown reason), in all case of MAS TCZ was continued.

## Conclusion

TCZ is the best choice among B at sJIA in Pts independently on the age of manifestation of disease, duration of disease, the number of active joints, the number of active systemic features, previous B. Careful monitoring provided an acceptable safety profile of TCZ in the pts with sJIA. TCZ was well tolerated, and the majority of AE were mild or moderate, reversible, and not treatment limiting.

## Disclosure of interest

None declared.

